# Effects of intraoperative Magnesium sulfate infusion on emergency agitation during general anesthesia in patients undergoing radical mastectomy: a randomized controlled study

**DOI:** 10.1186/s12871-023-02288-6

**Published:** 2023-09-26

**Authors:** Yan-hong Su, De-cai Luo, Yong Pang

**Affiliations:** Department of Anesthesiology, Affiliated Hospital of North Sichuan Medical College, Nanchong, Sichuan 637000 China

**Keywords:** Radical mastectomy, Magnesium sulfate, Emergency agitation

## Abstract

**Background:**

Emergency agitation is a common postoperative complication in patients under general anesthesia, which can lead to unpredictable damages such as shedding of drainage tube and bleeding from the wound. The purpose of the study is to investigate whether intraoperative infusion of Magnesium Sulfate reduces the incidence of emergency agitation (EA) in patients undergoing radical mastectomy, and to evaluate its safety and efficacy.

**Methods:**

A total of 70 patients were randomly assigned to two groups: the Magnesium group (M group) and the control group (C group). After a routine intravenous anesthetic induction, patients in the M group received a 30 mg/kg bolus of intravenous magnesium during the first hour and then a continuous infusion of 10 mg/kg ×h until the end of the surgery, patients in the C group received 0.9% saline at the same volume and rate. The sedation-agitation scale (SAS) and the visual analogue scale were used to assess agitation and pain, respectively.

**Results:**

Compared to the C group, the M group reduced the incidence of EA significantly (odds ratio 0.26, 95% confidence interval 0.09–0.71, P = 0.009). The postoperative pain score of the magnesium sulfate group(0(0,1)) was lower than that of the control group(2(0,3)) at T0 (P = 0.011). Additionally, the M group required a lower dosage of remifentanil during surgery compared to the C group(300.4 ± 84 versus 559.3 ± 184 µg, respectively, P<0.001).

**Conclusions:**

the intraoperative infusion of magnesium sulfate is a safe and effective method for reducing the incidence of emergency agitation in patients undergoing radical mastectomy.

**Trail registration:**

The study was registered in Chictr.org with the identifier: ChiCTR2300070595 on 18/04/2023.

## Background

Emergency agitation (EA) is a commonly observed adverse reaction in patients undergoing general anesthesia, which characterized by a.

sequence of sudden and complex actions, including excitement, agitation, hyperactivity of limbs and unconscious crying [1]. Although it typically within a short period after extubation (usually 5-10 min, and less than 30 min), it can pose potential risks to both patients and medical staff. Postoperative agitation not only leads to inadequate nursing resources but also prolongs hospital stay and increases hospital costs [2, 3]. Therefore, it is crucial to explore safe and effective approaches to reduce the incidence of EA in clinical practice. Clinical anesthesiologists have made great endeavors to decrease EA and have achieved significant success in this regard. Sedatives and analgesics are the most frequently used medications to administrate emergency agitation, such as opioids, dexmedetomidine, ketamine and magnesium sulfate [4, 5]. Magnesium sulfate is an NMDA receptor antagonist [6], which is increasingly being used as an adjuvant agent [7]. Studies have demonstrated that magnesium sulfate has been validly utilized to reduce the occurrence of EA in children [5], and it does not cause any side effects. Radical mastectomy is a frequently used surgical method for treating breast cancer [8]. However, it is a high-risk factor associated with surgery for EA [3]. According to some reports, the overall incidence of emergency agitation in adults ranges from 0.25 to 19% [9]. It is still unclear whether intravenous infusion of magnesium sulfate can reduce the incidence of EA during the early recovery stage of radical mastectomy in patients under general anesthesia. Therefore, the purpose of the current study was to evaluate the effects of intravenous infusion of magnesium sulfate on EA in female patients undergoing radical mastectomy and provide evidence for improving recovery and prognosis.

## Materials and methods

### Ethics and participants

This randomized controlled study was conducted at Affiliated Hospital of North Sichuan Medical College between January 2023 and February 2023, and was approved by the Ethics Committee of Affiliated Hospital of North Sichuan Medical College(2022ER494-1). All methods are in line with the relevant guidelines and regulations of the study. Written informed consent was obtained from all participants aged 18–65 years old who were scheduled for radical mastectomy and had an ASA physical status of I or II. The following were the exclusion criteria: hypertension, cardiac ischemia, history of cardiac conduction block, history of neuromuscular disease, pregnancy, chronic treatment with calcium channel blockers, liver dysfunction, diabetic neuropathy or known to hypersensitive to magnesium compounds. Patients who declined to participant were also excluded. All participants were randomly assigned to one of two parallel groups of equal size, with one group receiving intraoperative magnesium (the magnesium group) and the other group receiving saline (the control group).

### Randomization

Patients were randomly divided into the magnesium sulfate group (M group) and the control group (C group) by internet-based randomization software with a 1:1 ration, and the information of each patient was sealed in an envelope. On the day of surgery, the anesthesia nurse randomly selected an envelope to determine whether the patient in the M or C group. Data collection was performed by an anesthetic technician who did not participant in the study. Participants and investigators were blinded to group allocation.

### Anesthesia and intervention

Every subject had no premedication. On arrival at the operating room, ECG, pulse oximetry (S_P_O_2_), non-invasive blood pressure on the calf (NIBP, to facilitate surgery) and bispectral index (BIS,KeHui Medical Equipment International Trade (Shanghai) Co., Ltd.) were monitored. MAP and HR were documented as baseline values approximately 10 min after the patients arrived. Anesthesia induction was performed with propofol 2mk/kg(Corden Pharma Latina S.p.A) and fentanyl 3 µg/kg (Jiangsu Enuowa Pharmaceutical Co., Ltd.), followed by cis-atracurium 0.15 mg/kg (Nanjing Jianyou Biochemical Pharmaceutical Co., Ltd.) to paralyze the muscles. Tracheal intubation was performed when the anesthesia reached an appropriate depth, and mechanical ventilation was conducted with respiratory parameters adjusted to a suitable range. In the magnesium group, patients received a 30 mg/kg bolus of intravenous magnesium(Brilliant Pharmaceutical Co., Ltd.) within one hour after induction and then a continuous infusion of 10 mg/kg ×h until the end of the surgery, patients in the control group received 0.9% saline at the same volume and rate. Remifentanil (Jiangsu Enuowa Pharmaceutical Co., Ltd.) was titrated to provide analgesic effect for patients at a rate of 0.1ug/kg/min during the surgery. In the meanwhile, sevoflurane was titrated to maintain the mean arterial pressure within 20% of baseline, heart rate no less than 50bmp and the BIS value at 40–60. Atropine or vasopressor was administrated if the two values observed below maintenance range. Remifentanil was terminated while stitching the wound, and every patient received intravenous fentanyl 1 mg/kg as postoperative analgesia. At the end of the surgery, all other medications were disconnected.

### Data collection

Indicators, including EA, postoperative pain, as well as the means to manage them, were evaluated by a blinded investigator. EA was assessed using the Rikers Sedation Agitation scale [10, 11], which includes the following categories: (1) Unresponsive; (2) Very sedated; (3) Sedated: Difficult to arouse; (4) Calm, cooperative: Calm, easily aroused, follows commands;5. Agitated: Anxious or mildly agitated, becomes calm when verb instructions are provided;6. Very agitation: cannot remain calm, despite frequent verbal remainders of limits; 7. Dangerous agitation. The scale has been widely applied and has been proved to be both valid and reliable [12]. Postoperative pain was evaluated using the Visual Analogue Scale [13]. Tramadol (Grunenthal GmbH) was administred intravenously at a dose of 25 mg as a rescue analgesic when patients occurred agitation. Medication was only allowed after the evaluation of EA and pain scoring were completed to avoid any confusion regarding outcome measurements.

### Observation outcomes

After surgery, patients were transferred to the Post-Anesthesia Care Unit (PACU). The primary observation outcome was the incidence of emergency agitation 0 min (T0) arrived at the PACU, and the incidence of EA at 5(T1), 10(T2), 15(T3), 30(T4) minutes after arrival. The secondary outcomes included the VAS scores, the Rikers SAS scores, the consumption of remifentanil, reaction time (from sevoflurane disconnection to recovery of consciousness), extubation time (from sevoflurane disconnection to extubation) and tramodol comsuption. Any adverse effects related to the intravenous infusion of magnesium sulfate, such as arrythmia or neuromuscular paralysis and breathing difficulties, were also documented.

### Statistical analysis

The sample size of the study was calculated from the incidence of EA at 0 min entering the PACU of the preliminary experiment based on 15 participants each group, consequently, subjects (32 per each) were required to attained a power of 80% and a type I error of 0.05. To account for a potential 10% dropout rate, we enrolled 35 subjects each group.

Statistical analysis was performed by SPSS (ver.25.0, IBM Co., Armonk, NY, USA). For normally distributed data, means and standard deviation (mean ± SD) were used, and the difference was compared with the Student’ s t-test. For non-normally distributed data, median (interquartile distance) M (P_25_, P_75_) was used for results, and the difference was analyzed using the Mann-Whitney U test. Categorical data was expressed as percentages and analyzed using the Chi-square test or Fisher’s exact test. A P value less than 0.05 was considered statistically significant.

## Results

**Fig. 1:**
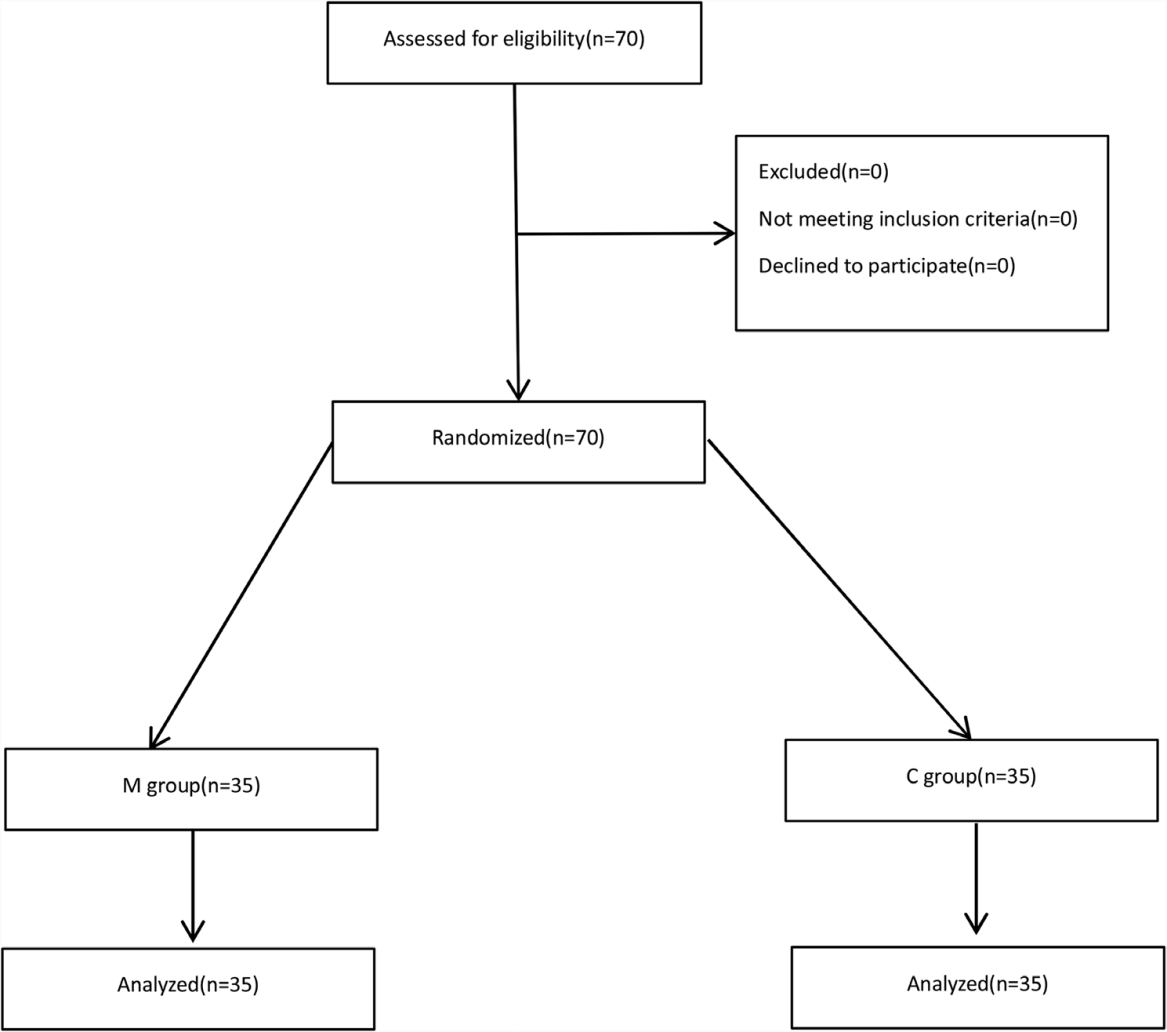
We enrolled 70 patients all together and 70 patients were assessed for eligibility, they were randomly alloeated to two groups: magnesium group (M Group. n = 35) and the control group (C Group. n = 35). none of them were be dropped out

In this study, we enrolled 70 patients all together who were assessed for eligibility and randomly allocated to two groups: magnesium group (M Group, n = 35) and the control group (C Group, n = 35) (Fig. 1).

None of the patients were dropped out for various reasons. Demographic characteristics are presented in Table 1. There were no statistically differences between the two groups in the basic data of patients such as age, height, weight, duration of anesthesia and duration of surgery. Reaction time, extubation time, the incidence of PONV and the mean end-tidal sevoflurane concentration were similar between the two groups (Table 2). However, it was found that intraoperative remifentanil consumption was different significantly between them (300.4 ± 84 versus 559.3 ± 184 µg in M group and C group, respectively, P<0.001)(Table 2). The total dose of rescue analgesics of two groups was comparable (P<0.001) (Table 2).

**Table 1:** Demographic characteristics

variable	M group(n = 35)	C group(n = 35)	P value
Age; years	52(48, 57)	54(48, 59)	0.374
Height; cm	154.4 ± 4.0	154.9 ± 6.1	0.727
Weight; kg	58.5 ± 6.1	57 ± 7.2	0.332
Duration of anesthesia; min	166(152,187)	154(138,183)	0.224
Duration of surgery; min	128(117, 145)	120(103, 150)	0.347

Categorical variables were represented as mean ± standard deviation, number

of patients (%) or median(range). PONV, postoperative nausea and vomiting. ET-sevo, the mean end-tidal sevoflurane concentration. ^*^P<0.05

**Table 2:** Perioperative measurements

variable	M group(n = 35)	C group(n = 35)	P value
Reaction time; min	10(9,12)	10(9,12)	0.967
Extubation time; min	12(10,13)	12(11,13)	0.528
PONV (n, %)	3(8.6)	5(14.3)	0.644
The consumption of remifentanil; µg	300.4 ± 84	559.3 ± 184	<0.001^*^
Dose of rescue analgesics; mg	0(0,25)	25(25,25)	<0.001^*^
ET-Sevo (vol, %)	1.7(1.5, 1.9)	1.7(1.6, 1.9)	0.609

Categorical variables were represented as mean ± standard deviation, number

of patients (%) or median(range). PONV, postoperative nausea and vomiting. ET-sevo, the mean end-tidal sevoflurane concentration. ^*^P<0.05

We assessed EA at various time points after admission to PACU and found that intraoperative infusion of magnesium sulfate significantly reduced the incidence of EA at T0 (odds ratio 0.26, 95% confidence interval 0.09–0.71, P = 0.009), T1 and T2(P = 0.039,P = 0.035),(Table 3). However, the incidence of EA was statistically similar at T3 and T4 between the two groups. EA was still present in one patients in the M group and four in the C group at T3, which indicated there was no significant different between them (1(2.8%) versus 4(11.4%) ,P = 0.197), no patients in either group showed EA at T4 (Table 3). The Rikers SAS scoring was also performed, the M group received patently lower scores compared to the C group at T0 (4(3,5)versus 5(4,5), P<0.001), T1and T2(P = 0.007, P = 0.027), (Table 3). The severity of EA at T3 (P = 0.579) and T4 (P = 0.645) were no significant difference between the two groups, which were similar to that the incidence of EA described above(Table 3). All patients were given certain analgesic before the end of the surgery, and postoperative pain was well controlled, resulting in relatively low overall scores (Table 4). In terms of the VAS scores, there was a statistically significant difference between the two groups at each assessment point (P<0.05 Table 4). No adverse effects associated with magnesium sulfate were observed.

**Table 3:** Postoperative emergency agitation at various points

	M group(n = 35)	C group(n = 35)	P value
Incidence of EA (n, %)			
T0	9(25.7)	20 (56)	0.009^*^
T1	6(16.7)	14(40)	0.039^*^
T2	2(5.7)	9(25.7)	0.035^*^
T3	1(2.8)	4(11.4)	0.197
T4	0	0	1
The Rikers SAS scoring			
T0 95%CI	4(3,5) 3.6,4.2	5(4,5) 4.5,4.9	<0.001^*^
T1 95%CI	4(3,4) 3.7,4.1	4(4,5) 4.1,4.5	0.007^*^
T2 95%CI	4(4,4) 3.8,4.1	4(4,5) 4.0,4.4	0.027^*^
T3 95%CI	4(4,4) 3.8,4.1	4(4,4) 3.8,4.2	0.579
T4 95%CI	4(4,4) 3.8,4.0	4(4,4) 3.9,4.0	0.645

Categorical variables were represented as mean ± standard deviation, number of patients(%), median(range) and 95% confidence interval. EA, emergency agitation. SAS, sedation and agitation scale. CI, confidence interval. ^*^P<0.05

**Table 4:** Postoperative pain profile within 30 min

VAS scores	M group(n = 35)	Control group(n = 35)	P value
T0	0(0,1)	2(0,3)	0.011^*^
T1	0(0,1)	1(0,2)	0.013^*^
T2	1(0,2)	2(0,3)	0.020^*^
T3	1(0,2)	2(2,3)	<0.001^*^
T4	2(1,2)	2(2,3)	<0.001^*^

Categorical variables were represented as mean ± standard deviation, number of patients (%), median(range) and 95% confidence interval. VAS, visual analogue scale. ^*^P<0.05

## Discussion

In the current study, it was turned out to be that intraoperative infusion of magnesium sulfate was beneficial to reduce the incidence of emergency agitation during general anesthesia in patients undergoing radical mastectomy. Meanwhile, this intervention was found to diminish the consumption of remifentanil during the surgery and alleviate postoperative pain.

The etiology of EA still remains unclear, and it may be the result of multiple factors working together. Possible major confounders may include the patient’s individual characteristics, type of surgery, inadequate analgesia, and rapid awaking from inhaled anesthesia drugs [14–16]. The occurrence of EA during the recovery period in this experiment is similar to that of the previous experiment [5]: there was no significant difference in the occurrence rate of EA at T15 and T30, but it was different from the other experiment [17], possibly due to differences in the observed subjects, surgical methods, measurement methods, etc. And the high prevalence of EA in our present study may be account for the feature of surgery and the use of inhalational anesthetic. Radical mastectomy is an independent surgery-related risk factor for EA, which may be connected with postoperative pain and excessive anxiety caused by the change of appearance of breast [2]. Before the surgery in our trail, all patients had no premedication, research has demonstrated that Preoperative avoidance of benzodiazepines resulted in a significantly lower incidence of emergence agitation compared to other similar studies [18].

The occurrence of EA of pediatric sevoflurane anesthesia has been well documented in literature [19]. The pathophysiological mechanism of postoperative agitation caused by sevoflurane anesthesia is complex, and there is currently no unified conclusion. Some studies suggested that rapid emergency from inhalation anesthetics has been deemed as one of the factors of EA: Sevoflurane significantly affects GABA metabolism in the central nervous system and has a strong inhibitory effect on the central nervous system [20]. When patients rapidly recover from seven fluorine methane, the inhibitory effect of GABA on the central nervous system weakens, and finally, agitation occurs during the awakening period. But the hypothesis has encountered a challenge: Propofol-based anesthesia appeared a significantly lower reduction of EA, with a same emergency profile [21]. Some scholars have confirmed that after anesthesia with sevoflurane, the concentration of glucose and lactate in the brain of the patient increases simultaneously, which may lead to agitation during the recovery period, and this may also be the mechanism behind it [22]. Animal study has shown that magnesium sulfate could inhibit lactic acid concentration in the brain, improve brain hemodynamics and alter electroencephalogram changes after cerebral ischemia [23, 24].

Magnesium sulfate is a traditional agent widely applied in. clinical practice, magnesium is not only used to reduce agitation during the awakening period, but also to protect the brain nerves and improve cognitive function [25]. Glutamate is an excitatory neurotransmitter that can activate NMDA receptors, enhance calcium ion influx, and exacerbate nerve damage. Magnesium sulfate is an NMDA receptor blocker that can reduce the release of excitatory amino acids, thereby reducing the toxic effects of cerebral ischemic damage on nerves. Magnesium sulfate has the potential for neuroprotection, which could explain the observed reduction in the incidence of agitation after the intraoperative of magnesium sulfate in this study.

Postoperative pain is an important cause of EA [26], but it cannot be considered the only reason for agitation during the awakening period. Research has found that even for the purpose of general anesthesia to facilitate pediatric magnetic resonance imaging, 47.6% of patients can experience agitation during the awakening period [27]. There is still controversy regarding the correlation between postoperative pain, magnesium sulfate, and EA so far. Weldon et al. indicated that EA could not be relieved effectively after patients with EA during recovery from general anesthesia are treated with analgesic [28]. A meta-analysis showed that fentanyl, α2 receptor agonists, and ketamine achieve the goal of reducing agitation during emergence from anesthesia through their sedative mechanisms rather than their analgesic mechanisms [29].

In recent years, there have been many studies on intravenous magnesium sulfate infusion. A previous study verified that magnesium sulfate reduced the total dose of remifentanil [23], which is consistent with the current study. The analgesic effect of intravenous magnesium sulfate infusion mainly manifests in two aspects: reducing postoperative pain and prolonging the duration of analgesia, but the dosage and administration methods of magnesium sulfate vary [30, 31]. In this present study, we observed that magnesium sulfate reduced postoperative pain, which is compatible with the theory mentioned above, but whether it prolonged the analgesic effect in patients receiving magnesium sulfate was not observed due to insufficient observation time.

When low blood pressure leads to inadequate perfusion, excessive accumulation of lactate is likely to cause increased agitation during the recovery period, and in severe cases, unconscious struggling may occur due to insufficient cerebral perfusion. Therefore, we need to maintain hemodynamic stability. During our anesthesia process, we monitor the BIS value to ensure that the patient is at an appropriate depth of anesthesia, and monitor the end-tidal carbon dioxide concentration to maintain it at 35–45, in order to reduce the risk of cerebral hypoperfusion.in addition, Magnesium sulfate can maintain hemodynamic stability, which may be due to magnesium ion blocking calcium channels and inhibiting the release of catecholamines from the adrenal medulla and peripheral adrenergic nerve endings [32].

When selecting an appropriate drug, the advantages and side effects of the drug in clinical practice should be fully considered. Although opioids have been reported to reduce agitation during emergence from anesthesia, they often cause adverse reactions such as delayed respiratory depression, nausea, and vomiting after surgery [33], which are not conducive to the prognosis of patients. Magnesium sulfate can maintain hemodynamic stability and reduce the incidence of common perioperative complications. In addition, to our knowledge, magnesium has not yet been used to prevent or alleviate emergency agitation after radical surgery for breast cancer.

However, limitations of the current study should be observed: Firstly, it was a single-center clinical study with a relatively small number of subjects, so larger sample sizes and multi-center studies are needed to provide further support for the conclusion. Secondly, postoperative pain scores were only recorded within 30 min after the surgery, so it is unknown whether there is a significant difference between the two groups and whether analgesics are applied after 30 min. Lastly, Blood magnesium concentration was not monitored in each group before and after the surgery, and the dosage of magnesium sulfate was based on a previous study, so further investigations are needed to determine the optimal dose schedule of magnesium sulfate.

## Conclusion

The intraoperative infusion of magnesium sulfate is a safe and effective method for reducing the incidence of emergency agitation in patients undergoing radical mastectomy, in the meanwhile, the administration of intraoperative magnesium sulfate infusion can decrease the remifentanil consumption and postoperative pain. Moreover, it does not appear to be connected with any increased postoperative side-effects.

## Data Availability

The datasets used and analyzed during the current study are available from the corresponding author on reasonable request.
